# Realizing *p*-type NbCoSn half-Heusler compounds with enhanced thermoelectric performance via Sc substitution

**DOI:** 10.1080/14686996.2020.1726715

**Published:** 2020-02-25

**Authors:** Ruijuan Yan, Wenjie Xie, Benjamin Balke, Guoxing Chen, Anke Weidenkaff

**Affiliations:** a Department of Materials Science, Technical University of Darmstadt, Darmstadt, Germany; b Fraunhofer Research Institution for Materials Recycling and Resource Strategies IWKS, Alzenau, Germany

**Keywords:** *P*-type NbCoSn, thermoelectric materials, half-Heusler compound, 210 Thermoelectronics, Thermal transport, insulators, 206 Energy conversion, transport, storage, recovery

## Abstract

*N*-type half-Heusler NbCoSn is a promising thermoelectric material due to favourable electronic properties. It has attracted much attention for thermoelectric applications while the desired *p*-type NbCoSn counterpart shows poor thermoelectric performance. In this work, *p*-type NbCoSn has been obtained using Sc substitution at the Nb site, and their thermoelectric properties were investigated. Of all samples, Nb_0.95_Sc_0.05_CoSn compound shows a maximum power factor of 0.54 mW/mK^2^ which is the highest among the previously reported values of *p*-type NbCoSn. With the suppression of thermal conductivity, *p*-type Nb_0.95_Sc_0.05_CoSn compound shows the highest measured figure of merit *ZT* = 0.13 at 879 K.

## GRAPHICAL ABSTRACT

**Figure:**
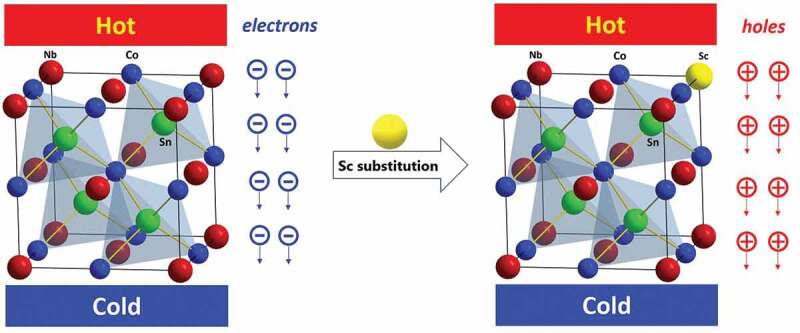

## 1. Introduction

Thermoelectric (TE) devices can directly convert waste heat into electricity via the Seebeck effect, which is a promising reliable alternative to mechanical converters if the efficiency can be improved [1]. This could become a pathway to more sustainable energy converters in times of energy consumption [2–5]. However, up to now the application of thermoelectric devices is limited by the low energy conversion efficiency *η*, which is essentially decided by the materials’ dimensionless figure of merit *ZT: ZT* = *S*^2^*σT*/*κ* (*κ* =* κ*_c_ + *κ*_L_), where *S* is the Seebeck coefficient, *σ* is the electrical conductivity, *κ* is the total thermal conductivity (*κ*_c_ and *κ*_L_ are the carrier and lattice components of *κ*, respectively), and *T* is the absolute temperature [6,7]. Thus, the combination of high *S, σ* and low *κ* is desirable for a large *ZT*. Unfortunately, these thermoelectric parameters are strongly coupled via carrier concentration and mobility [8–10], making it difficult to optimize one single parameter without altering the others. Therefore, it has been quite challenging to develop highly efficient thermoelectric materials. The strategic goals of TE research are to discover new materials with high TE performance and/or improve the performance of the existing well-known materials, such as SiGe alloy [10], PbTe [11], chalcogenides [12], skutterudites [13,14], clathrates [15,16], Zintl phases [17] as well as full/half-Heusler compounds [18,19] by band engineering and carrier filtering effect [20–22], phonon engineering [23,24], reducing the dimension of materials [25], or spin fluctuation [26].

Due to the excellent electrical and mechanical properties, high-temperature stability, and possibility to use non-critical elements, half-Heusler (HH) compounds have become attractive candidates for thermoelectric application [27–31]. This compound has a general formula *XYZ* (*X, Y* = transition metals, *Z* = main group elements), crystallizing in cubic *C*1*_b_* structure, $F\overset{ˉ}{4}3m$ space group [32,33]. An empirical rule proposed by Mahan and Sofo [34] states that the best thermoelectric performance is found with materials whose band gap is about 10*k*_B_*T*_0_, where *k*_B_ is the Boltzmann constant and *T*_0_ is the operating temperature of the device. NbCoSn is one of HH thermoelectric compounds with a band gap of 0.987 eV [35], and such a band gap fulfils the ‘10*k*_B_*T*_0ʹ_ rule. In addition, the small electronegative difference between Co (1.88) and Sn (1.96) ensures larger carrier mobility. Therefore, NbCoSn is a promising mid-high temperature thermoelectric material. Unsubstituted NbCoSn is an intrinsically *n*-type semiconductor. In 2006 Ono et al. [36] investigated the Sb and Ti substituted *n*-type NbCoSn and the highest *ZT* of 0.3 was achieved for the Nb_0.99_Ti_0.01_CoSn_0.9_Sb_0.1_ at 850 K. A decade later, He et al. [37] synthesized *n*-type NbCoSn_1-*x*_Sb*_x_* samples by arc melting combined with ball milling and hot-pressing processes, and the highest *ZT* reached ~0.6 at 1000 K for NbCoSn_0.9_Sb_0.1_. Generally, a TE device needs not only high *ZT* in *n*-type and *p*-type materials, but the *n*-type and *p*-type materials should have similar compositions and thus comparable mechanical properties and thermal expansion coefficient. For instance, *n*-type *M*Ni(Sn,Sb) [38] and *p*-type *M*Co(Sb,Sn) [39,40] (*M* = Zr and Hf) compounds fulfil such criteria, and the thermoelectric module made of them reaches a record-high conversion efficiency of 12.4% with a temperature difference of 698 K [41]. To fabricate *p-n* NbCoSn couple, a *p*-type NbCoSn based compound is needed to match with the developed *n*-type NbCoSn compounds. However, few efforts have been devoted to investigating *p*-type NbCoSn. To the best of our knowledge, Ferluccio et al. [42] first realized *p*-type NbCoSn via substituting Ti and Zr at Nb site, and the maximum *ZT* value for *p*-type Nb_0.8_Zr_0.2_CoSn is only 0.03 at ~790 K. Obviously, the *ZT* value of *p*-type Nb_0.8_Zr_0.2_CoSn compounds is much inferior to those of *n*-type counterparts. Therefore, it is crucial to identify new *p*-type dopants and further improve the thermoelectric performance of *p*-type NbCoSn.

In this work, the NbCoSn compound has been prepared through arc melting followed by annealing processes, and then Sc, chosen as a *p*-type dopant, is substituted at Nb site to obtain Nb_1-*z*_Sc*_z_*CoSn (*z* ≤ 0.1). There are two main reasons for choosing Sc as a *p*-type dopant: (1) The substitution of Nb (III B) by Sc (V B) can create more holes in this compound and further achieve a *p*-type NbCoSn. (2) The larger mass fluctuation between Nb (92.91 g/mol) and Sc (44.96 g/mol) can strengthen defect and alloying phonon scattering which can significantly suppress the lattice thermal conductivity. It is found that Nb_1-*z*_Sc*_z_*CoSn compound possesses *p*-type conducting behaviour when Sc = 0.05. The highest power factor of this *p*-type compound is 0.54 mW m^−1^ K^−2^, which is 230% higher than the reported value of Nb_0.8_Zr_0.2_CoSn [42]. As a result, a peak *ZT* of ~0.13 is achieved.

## 2. Experimental details

Nb_1-*z*_Sc*_z_*CoSn (*z* ≤ 0.1) compounds were prepared by arc-melting stoichiometric amounts of the elements Nb (wire, 99.999%), Sc (piece, 99.99%), Co (bulk, 99.999%), Sn (shot, 99.999%) in Ar atmosphere. The ingots were melted several times with flipping twice over each time to ensure homogeneity. The obtained ingots were sealed into evacuated quartz tubes, annealing at 1173 K for 7 days. And then the bars and pellets for measurements were prepared by cutting these annealed ingots.

The crystal structures of samples were investigated by powder X-ray diffraction (PXRD) on a Rigaku diffractometer (Rigaku, Japan) using Cu Κ_α_ radiation (*λ*_0_ = 1.5418 Å). The microstructures of polished samples were characterized by scanning electron microscopy (SEM; Zeiss Gemini, Germany), and the phase compositions were analyzed by energy-dispersive X-ray spectroscopy (EDX; Bruker, Germany). Electrical transport properties (Seebeck coefficient and electrical conductivity) were simultaneously measured by a ZEM-3 instrument (Ulvac-Riko, Japan) under He atmosphere from 300 to 900 K. The measurement errors for all samples are around ±3% (electrical conductivity) and ±5% (Seebeck coefficient). The Hall carrier concentration *p*_H_ (*n*_H_) and mobility *μ*_H_ were calculated via *p*_H_ = 1/*eR*_H_ (*n*_H_ = ﹣1/*eR*_H_) and *μ*_H_ = *σR*_H_, where *e* is unit charge and *R*_H_ is the Hall coefficient measured by commercial Physical Properties Measurement System (PPMS; Quantum Design, USA) under magnetic fields from −5.2 T to 5.2 T. The thermal conductivity was calculated by the formula *κ* = *DC*_p_*d_s_*, where *D* is thermal diffusivity measured by laser flash instrument (NETZSCH, LFA457, Germany) by coating all samples with a thin layer of graphite to minimize emissivity errors (the actual measurement error is about 3%), *C*_p_ is specific heat derived by temperature-dependent heat data using the differential thermal analyzer (NETZSCH, DSC204F1, Germany), and *d_s_* is the samples’ density estimated by the Archimedes method. The relative densities of these samples are about 95%.

## 3. Results and discussion

### 3.1. Phase and microstructure

**Figure 1.**
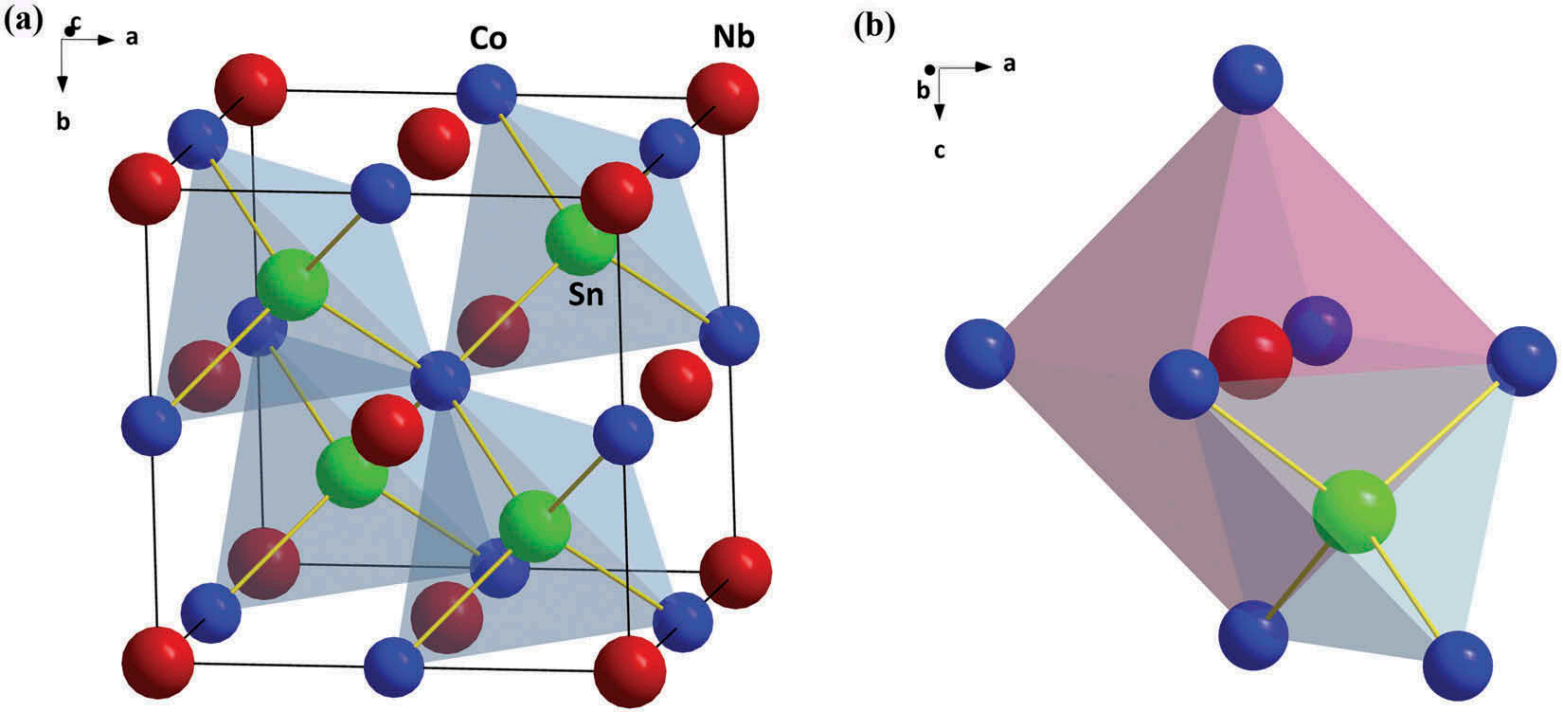
The crystal structure of NbCoSn (a) and the coordination environment of Nb and Sn (b)

The cubic NbCoSn crystal structure is shown in Figure 1. The element Nb (4a site) and Co (4b site) frame the NaCl sublattice with octahedral coordination, leaving the all tetrahedral central sites (4c site) to the element Sn, but only half of the 4c site is occupied by Sn, forming this half-Heusler compound. Simply, by applying the Zintl chemistry concept [43], this crystal structure can be described into an anionic framework [CoSn]^5-^ formed by the tetrahedral coordination of Co and Sn, and an electropositive Nb^5+^ filled in the octahedral voids formed by these tetrahedral frameworks.

**Figure 2.**
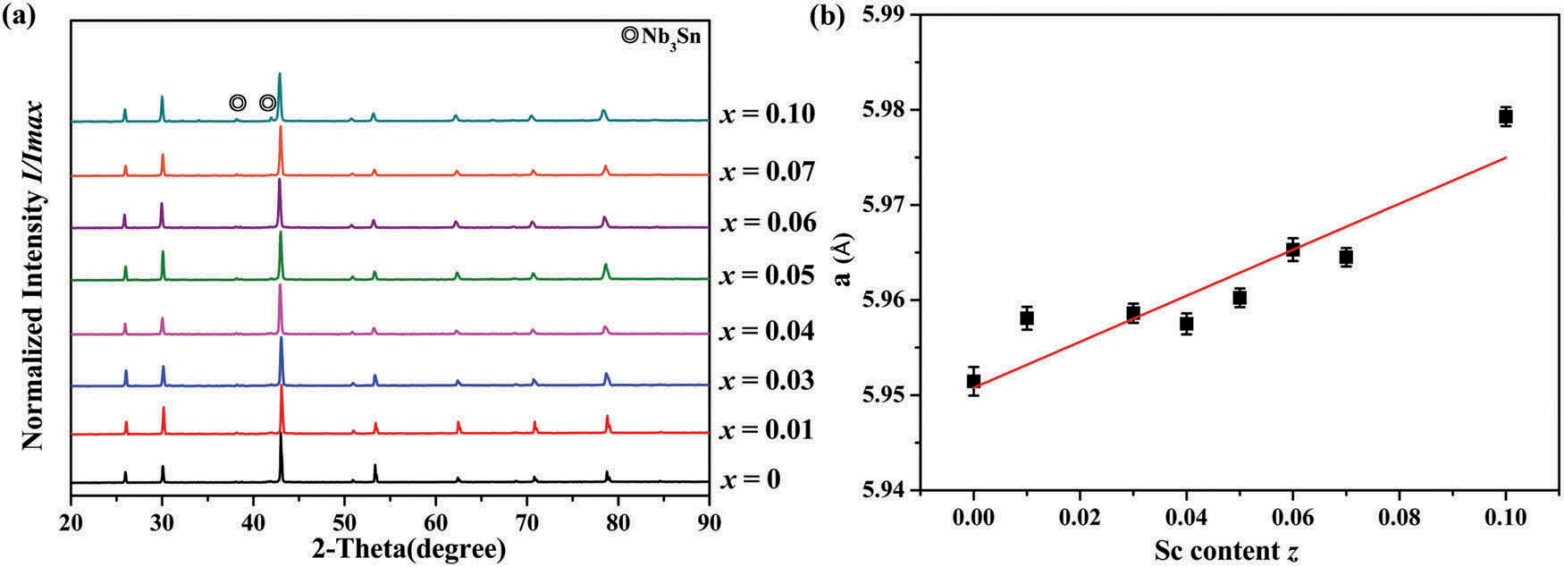
(a) PXRD patterns and (b) cell parameters of Nb_1-*z*_Sc*_z_*CoSn samples

PXRD patterns of Nb_1-*z*_Sc*_z_*CoSn (*z* = 0, 0.01, 0.03, 0.04, 0.05, 0.06, 0.07, 0.10) samples are shown in Figure 2(a). The diffraction peaks of all the samples can be indexed to MgAgAs cubic crystal structure despite some minor Nb_3_Sn impurity phases, indicating all samples possess the HH phase. As it is visible from Figure 2(b), the unit cell parameter calculated via PowderCell [44] software increases with the increasing Sc content. Since the ionic radius of Sc^3+^ (0.87 Å) is larger than that of Nb^5+^ (0.74 Å) [45], the observed lattice expansion indicates Nb^5+^ is substituted by Sc^3+^.

**Figure 3.**
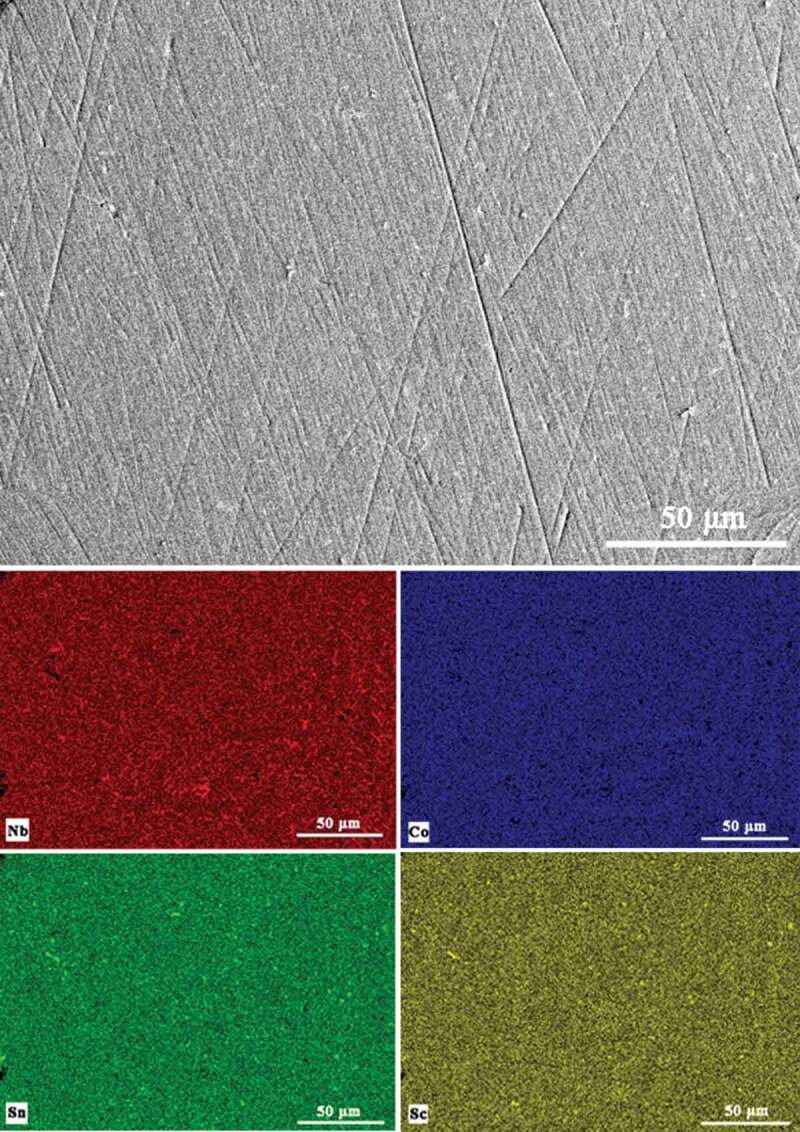
The secondary electron image and the elemental distribution maps of Nb_0.95_Sc_0.05_CoSn

The phase purity and elemental distribution were further examined by SEM combined with EDX mapping. Figure 3 shows the secondary electron image and the EDX maps of a polished Nb_0.95_Sc_0.05_CoSn sample, which indicates no obvious phase segregation and uniform elemental distribution on this scale. Table 1 summarizes the actual chemical compositions of all prepared samples analyzed by EDX, and it shows that the actual chemical compositions are close to the nominal compositions.

**Table 1.** Nominal, actual compositions by EDX and measured densities of Nb_1-*z*_Sc*_z_*CoSn

Nominal	EDX results	*d_s_* (g/cm^3^)
Nb_0.99_Sc_0.01_CoSn	Nb_0.98_Sc_0.01_Co_1.05_Sn_0.96_	8.49
Nb_0.97_Sc_0.03_CoSn	Nb_0.97_Sc_0.03_Co_1.06_Sn_0.94_	8.46
Nb_0.96_Sc_0.04_CoSn	Nb_0.98_Sc_0.04_Co_1.03_Sn_0.95_	8.03
Nb_0.95_Sc_0.05_CoSn	Nb_0.97_Sc_0.045_Co_1.03_Sn_0.95_	8.39
Nb_0.94_Sc_0.06_CoSn	Nb_0.94_Sc_0.053_Co_1.05_Sn_0.95_	8.00
Nb_0.93_Sc_0.07_CoSn	Nb_0.93_Sc_0.067_Co_1.05_Sn_0.96_	8.37
Nb_0.90_Sc_0.10_CoSn	Nb_0.95_Sc_0.09_Co_1.03_Sn_0.94_	8.27

### 3.2. Electrical transport properties

**Figure 4.**
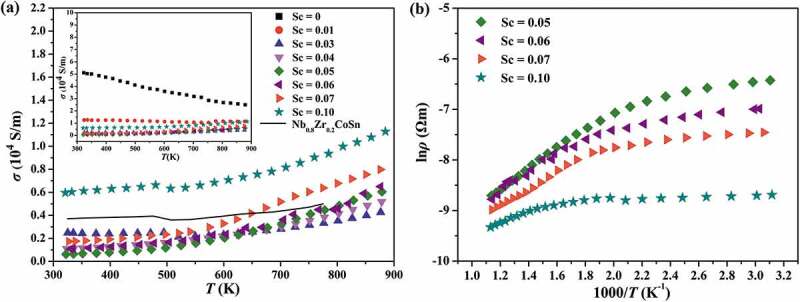
Temperature dependence of the electrical transport properties of Nb_1-*z*_Sc*_z_*CoSn (a) electrical conductivity (b) ln*σ vs*. 1000/*T* plot

Figure 4 shows the electrical transport properties of Nb_1-*z*_Sc*_z_*CoSn at different temperatures. For comparison, the literature data of Nb_0.8_Zr_0.2_CoSn [42] are also plotted (black line). As displayed in Figure 4(a), the electrical conductivity (*σ*) of unsubstituted NbCoSn compound decreases with the rise of temperature, showing a metal-like conducting behaviour. While, after substituting Sc at Nb site, the *σ* gradually increases with increasing temperature, indicating a semiconductor behaviour. Besides, with the Sc concentration up to 0.05, the *σ* significantly decreases from 5.0 × 10^4^ S/m (NbCoSn) to 0.07 × 10^4^ S/m (Nb_0.95_Sc_0.05_CoSn) at room temperature and then goes up to ~0.6 × 10^4^ S/m with further increasing Sc concentration to 0.1 (Nb_0.9_Sc_0.1_CoSn). In addition, the band gap can be obtained from the slope of ln*σ vs*. 1000/*T* curve (shown in Figure 4(b)) using the Arrhenius equation

(1) $$\rho =\rho_{0}exp\left(E_{g}/2k_{B}T\right)$$

where *ρ_0_* is a constant. The calculated band gap values are 0.35 eV, 0.33 eV, 0.29 eV and 0.20 eV for *p*-type Nb_0.95_Sc_0.05_CoSn, Nb_0.94_Sc_0.06_CoSn, Nb_0.93_Sc_0.07_CoSn and Nb_0.90_Sc_0.10_CoSn respectively. For the Nb_1-*z*_Sc*_z_*CoSn compounds with *z* > 0.04, the slope of *σ-T* changes above 500 K and it can be explained by the impact of intrinsic conduction.

**Figure 5.**
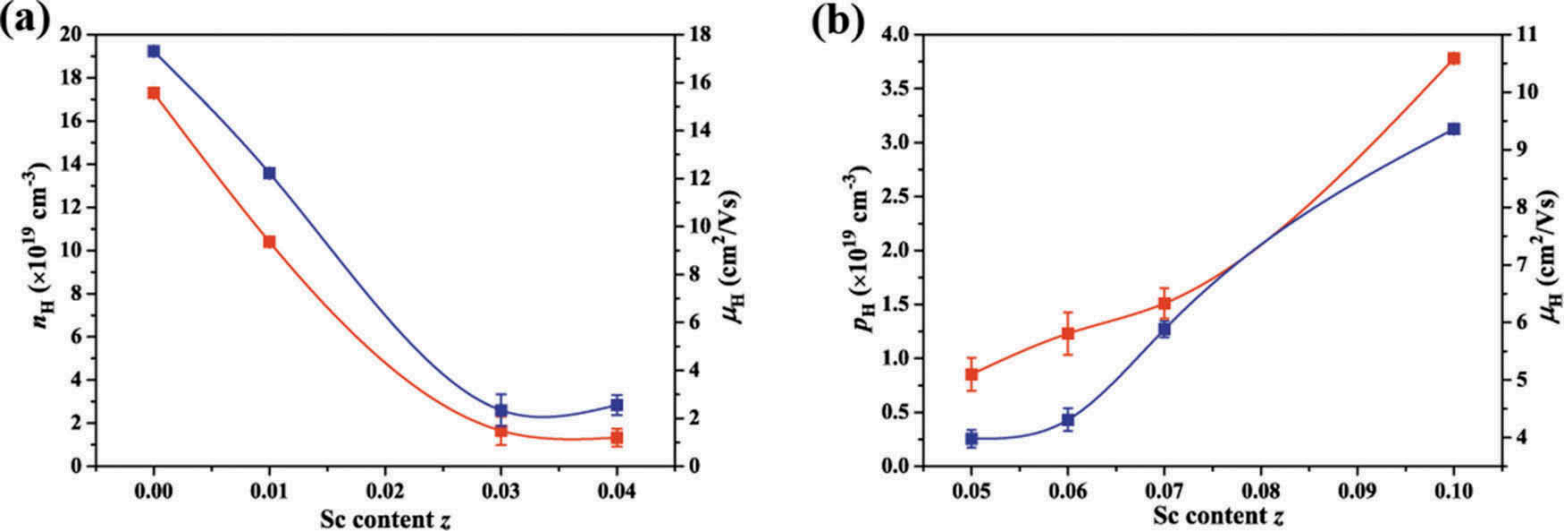
The carrier concentration and carrier mobility of Nb_1-*z*_Sc*_z_*CoSn samples at room temperature (a) *n*-type, (b) *p*-type

To further understand the conduction mechanism, the Hall coefficient at room temperature was measured and the calculated charge carriers concentration and mobility are presented in Figure 5(a,b). The electron concentration *n*_H_ for NbCoSn is 17 × 10^19^ cm^−3,^ which is on the same order of magnitude as compared to the value reported by He et al. (~24 × 10^19^ cm^−3^) [37]. Moreover, the *n*_H_ decreases remarkably with the Sc content reaching 0.04. For *z* = 0.05, holes become the dominant carriers, and the hole concentration *p*_H_ increases gradually from 0.8 × 10^19^ cm^−3^ to 3.7 × 10^19^ cm^−3^ with the Sc content increasing from 0.05 to 0.10. Therefore, Sc is obviously an effective hole (*p*-type) dopant since it generates acceptor level near the top of the valence band and shifts the Fermi level toward the valence band, resulting in an increase of hole concentration. As for the carrier mobility *μ*_H_, the trend is similar to that of carrier concentration. Therefore, the *σ* decreases obviously and then increases slightly with increase of Sc content.

**Figure 6.**
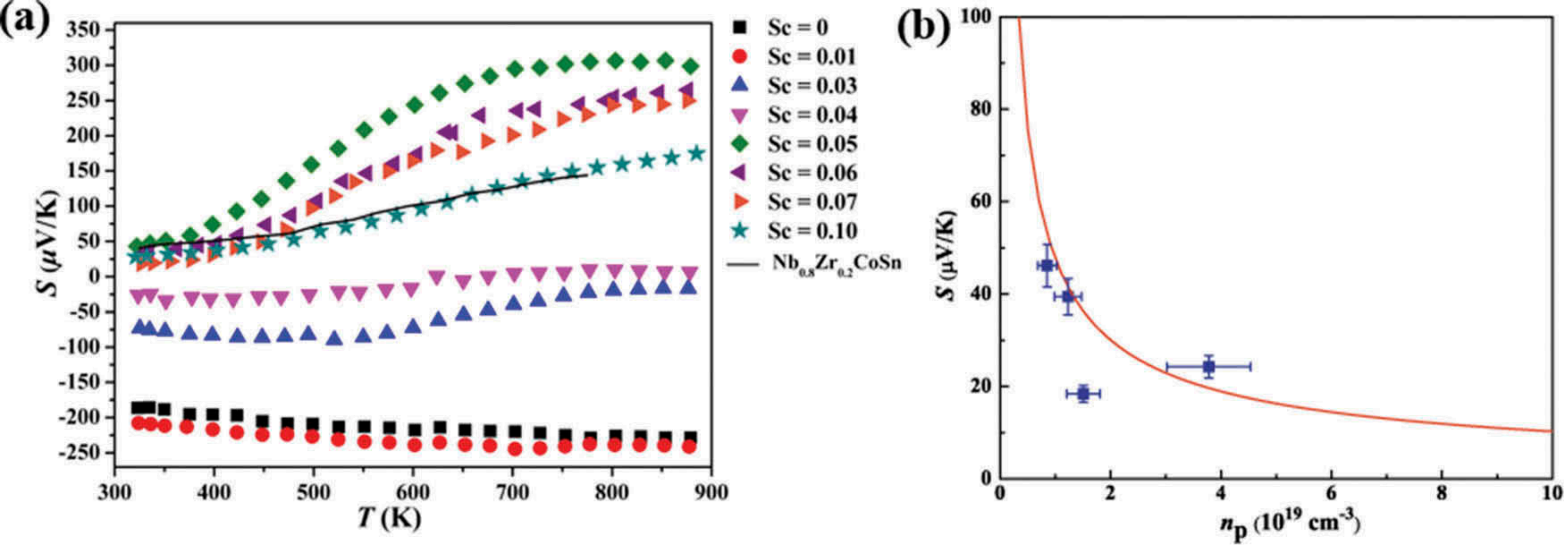
Temperature dependence of the Seebeck coefficient (a) and the Seebeck coefficient versus the carrier concentration (*p*-type) (b) of Nb_1-*z*_Sc*_z_*CoSn samples

The temperature dependence of Seebeck coefficient (*S*) in Nb_1-z_Sc_z_CoSn is shown in Figure 6(a). The value of *S* for unsubstituted NbCoSn is −185 *μ*V/K at room temperature, showing that NbCoSn is apparently an *n*-type semiconductor. With Sc content increasing, the *S* of Nb_1-*z*_Sc*_z_*CoSn changes gradually from negative to positive with increasing of Sc content, which matches very well with the Hall measurements. The peak value of *S* reaches ~306 *μ*V/K at 850 K for the Nb_0.95_Sc_0.05_CoSn sample, and it is much higher than that (150* μ*V/K) of Nb_0.8_Zr_0.2_CoSn. The single parabolic band (SPB) model is usually used to analyze the transport properties of half-Heusler compounds [46]. Assuming electron conduction occurs within an SPB, the Seebeck coefficient of a non-degenerate semiconductor is related to the effective mass m*, carrier concentration *p_H_* and scattering parameter *λ* via

(2) $$S=\frac{k_{B}}{e}\left\{2+\lambda +ln\left[\frac{2{\left(2\pi m^{*}k_{B}T/h^{2}\right)}^{3/2}}{p_{H}}\right]\right\}$$

where *e* is the elementary charge and *h* is the Planck constant [47]. For the NbCoSn compound, we assume that acoustic phonon scattering is the predominant scattering mechanism, thus *λ* = 0. According to the measured *S* and *p*_H_, the m* = 0.11*m*_e_ is obtained. With the m* = 0.11*m*_e_ and the Equation (2), we can plot *S* at 300 K as a function of *p_H_*, a plot well-known as a ‘Pisarenko relation’. As shown in Figure 6(b), the red line is the calculated Pisarenko plot, and the blue dots represent measured data of Nb_1-*z*_Sc*_z_*CoSn compounds. Most of the data lie on the calculated line, except for that of Nb_0.93_Sc_0.07_CoSn compound. The reason for such an exception is not clear yet. It is suspected that the second phase or/and the deviation of the composition may be responsible for such an exception.

**Figure 7.**
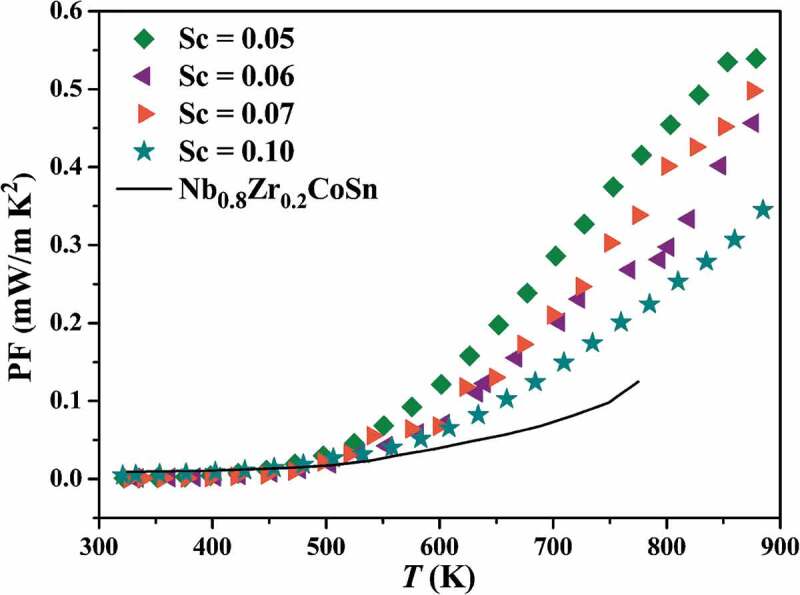
Temperature dependence of the power factor of Nb_1-*z*_Sc*_z_*CoSn

Accordingly, the power factor (PF) was calculated via PF = *S*^2^σ and is shown in Figure 7. The highest PF of 0.54 mW/mK^2^ is achieved for Nb_0.95_Sc_0.05_CoSn compound mainly due to its high *S*, and it is 3 times higher than that of *p*-type Nb_0.8_Zr_0.2_CoSn (0.125 mW/mK^2^) [42]. However, the PF of Nb_0.95_Sc_0.05_CoSn is still much lower than the state of the art *p*-type HH compounds, such as (Ti/Hf)Co(SbSn) [39] and NbFeSb [48]. Therefore, much more effort must be devoted to optimizing the carrier concentration of *p*-type NbCoSn compounds.

### 3.3. Thermal transport properties

**Figure 8.**
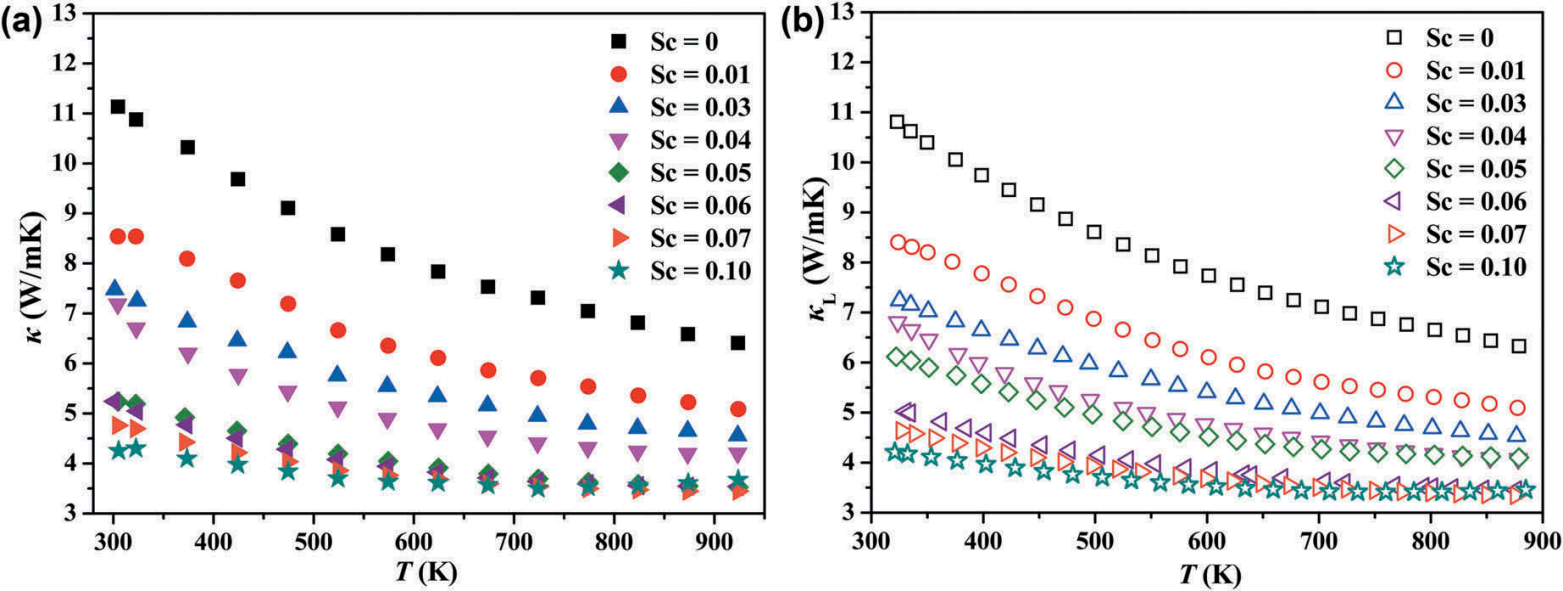
The temperature dependence of total thermal conductivity *κ* (a) and lattice thermal conductivity *κ*_L_ (b)

The temperature dependence of total thermal conductivity (*κ*) and lattice thermal conductivity (*κ*_L_) are displayed in Figure 8. The *κ*_c_ is calculated by using the Wiedemann-Franz law: *κ*_c_ =_ _*LσT*, where *L* is Lorenz number estimated by Fermi integral, and then *κ*_L_ is derived from the value subtracting the carrier component *κ*_c_ from the total thermal conductivity. Because of the low electrical conductivity, the calculated *κ*_c_ is much lower than *κ*_L_. In other words, for Nb_1-*z*_Sc*_z_*CoSn compounds *κ*_L_ ≈ *κ*. As shown in Figure 8(b), the *κ*_L_ of unsubstituted NbCoSn is ~10.8 W/mK at room temperature, and this value is similar as compared to the result reported by Ferluccio et al. [42]. After substituting Sc, the room temperature *κ*_L_ decreases dramatically to 4.2 W/mK for Nb_0.9_Sc_0.1_CoSn, where a reduction of 60% is achieved after Sc substitution. Such a significant reduction mainly ascribes to the point defect scattering due to the substantial atomic mass difference (mass fluctuation) and interatomic coupling force differences (strain field fluctuation) between Nb and Sc, thereby giving rise to the reduction of *κ*_L_, especially at room temperature.

To explain the reduction of *κ*_L_ in terms of the phonon scattering mechanisms, the lattice thermal conductivity of Nb_1-*z*_Sc*_z_*CoSn can be evaluated via Debye-Callaway model [49]:

(3) $$\kappa_{L}=\frac{k_{B}}{2\pi^{2}\nu_{s}}(\frac{2\pi k_{B}T}{h}{)}^{3}\int_{0}^{\theta_{D}/T}\tau_{C}\frac{x^{4}e^{x}}{{\left(e^{x}-1\right)}^{2}}dx$$

where *x* is the reduced frequency (*x* = *hω*/2π*k_B_*T), *ω* is the phonon angular frequency, *v_s_* is the sound speed, h is the Planck constant, *θ_D_* is the Debye temperature, and $\tau_{C}$ is the combined phonon relaxation time. The literature data [42] of *θ_D_* = 361 K and *v_s_* = 3141 m/s for NbCoSn are used in Nb_1-*z*_Sc*_z_*CoSn compounds as a good approximation. We assume all phonon scattering processes, including point-defect scattering, boundary scattering, Umklapp scattering, and phonon-free-electron scattering can occur in parallel and thus each adds to the process according to the Matthiessen’s rule, then the $\tau_{C}$ can be formulated in Equation (4)

(4) $$\tau_{C}^{-1}=\tau_{PD}^{-1}+\tau_{B}^{-1}+\tau_{U}^{-1}+\tau_{pe}^{-1}$$

where $\tau_{PD}$, $\tau_{B}$, $\tau_{U}$, and $\tau_{pe}$ are phonon-point-defect scattering, phonon-boundary scattering, phonon-phonon Umklapp scattering and phonon-free-electron scattering relaxation times, respectively. The $\tau_{PD}$ can be obtained through:

(5) $$\tau_{PD}^{-1}=\tau_{S}^{-1}+\tau_{M}^{-1}=\frac{V\omega^{4}}{4\pi v_{s}^{3}}\left(\Gamma_{S}+\Gamma_{M}\right)$$

where $\tau_{S}$ and $\tau_{M}$ are relaxation times of the phonon-point-defect scattering processes due to strain and mass field fluctuations, *V* is the volume per atom, $\Gamma_{S}$ and $\Gamma_{M}$ are the disorder scattering parameters due to strain and mass field fluctuations [50]. The experimental disorder scattering parameters *Γ_expt_* ($\Gamma_{expt}=\Gamma_{S}+\Gamma_{M})$ can be obtained by

(6) $$\Gamma_{exp}=\frac{hv_{s}^{2}u^{2}}{\pi^{2}\theta_{D}V}\times \frac{1}{\kappa_{L}^{P}},\;and\;\frac{\kappa_{L0}}{\kappa_{L0}^{P}}=\frac{tan^{-1}\left(u\right)}{u}$$

where *u* is the disorder scattering parameter,$\kappa_{L0}$ is the lattice thermal conductivity of the crystal with the disorder, and $\kappa_{L0}^{P}$ is the lattice thermal conductivity of the crystal without disorder [51]. The disorder scattering parameters of Nb_1-*z*_Sc*_z_*CoSn compounds calculated according to Equation (6) are listed in Table 2.

**Table 2.** The lattice thermal conductivity ***κ_L_***, disorder scattering parameter ***u***, disorder scattering parameters ***Γ*_expt._**

Composition	*κ*_L_	*u*	Γ_expt_
NbCoSn	10.8		
Nb_0.99_Sc_0.01_CoSn	8.4	1.03	0.0025(4)
Nb_0.97_Sc_0.03_CoSn	7.2	1.44	0.0049(8)
Nb_0.96_Sc_0.04_CoSn	6.8	1.61	0.0062(7)
Nb_0.95_Sc_0.05_CoSn	6.1	1.93	0.0089(8)
Nb_0.94_Sc_0.06_CoSn	5.0	2.59	0.016(1)
Nb_0.93_Sc_0.07_CoSn	4.6	2.88	0.019(9)
Nb_0.90_Sc_0.10_CoSn	4.2	3.27	0.025(6)

**Figure 9.**
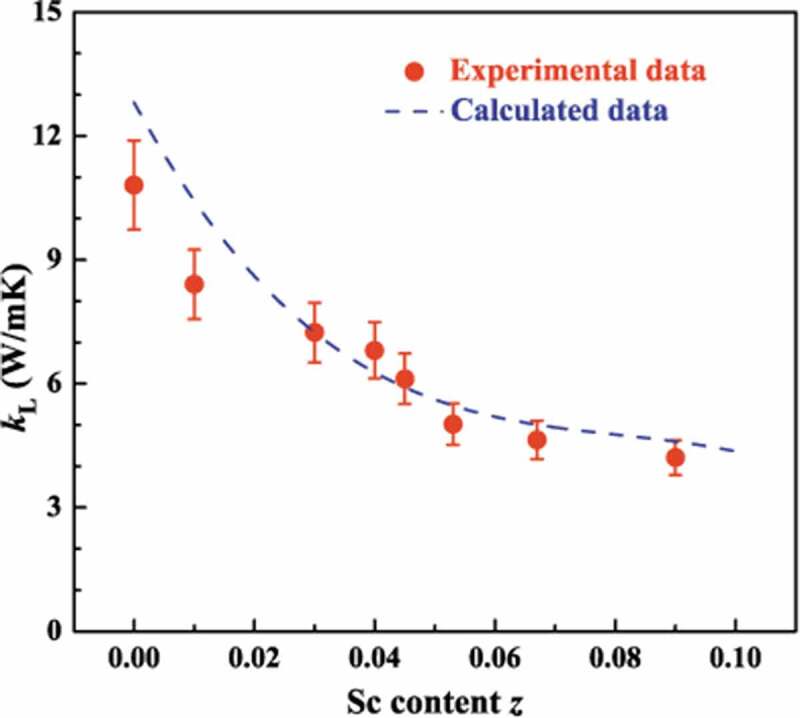
Comparison of experimental and calculated lattice thermal conductivities at 300 K for Nb_1-*z*_Sc*_z_*CoSn compounds

For the phonon-boundary scattering, $\tau_{B}$ is independent of temperature and phonon frequency, and it can be described as $\tau_{B}^{-1}=v_{s}/d$, where *d* is the grain size of the bulk sample. For the Umklapp scattering, $\tau_{U}$is dependent on temperature and phonon frequency and can be described as [52]

(7) $$\tau_{U}^{-1}\approx \frac{h\gamma^{2}}{2\pi Mv_{s}^{2}\theta_{D}}\omega^{2}Texp\left(-\theta_{D}/3T\right)$$

where γ is the Grüneisen constant and *M* is the average atomic mass of the crystal. For the phonon-free-electron scattering process [53], in the case of high carrier concentration $\tau_{pe}$ can be described as

(8) $$\tau_{pe}^{-1}=\frac{4\pi^{2}E_{def}^{2}m{*}^{2}\omega }{h^{3}d_{s}v_{l}}$$

where $E_{def}$ is the deformation potential, $d_{s}$ is the sample’s density, and $v_{l}$ is the longitudinal sound velocity. With the reference values of physics parameters (Table S1 in Supplementary Information), the lattice thermal conductivity of Nb_1-*z*_Sc*_z_*CoSn can be calculated by Equation (3) and the results are presented in Figure 9. In the calculation, we assume that Sc substitution does not significantly affect the basic physics parameters, such as *θ_D_*, γ, *E_def_* and $v_{s}$, so the major variable parameter is $\Gamma_{expt}$. In such a case, Sc substitution mainly alters the $\tau_{PD}$. Generally, the calculated *κ*_L_ matches with the experimental values, implying that calculations based on Callaway-Debye model can give a rough prediction to the *κ*_L_ of NbCoSn system. In short, at room temperature the reduction of *κ*_L_ of Nb_1-*z*_Sc*_z_*CoSn compounds is mainly due to that the Sc substitution induces strong point defect phonon scattering.

### 3.4. Figure of merit

**Figure 10.**
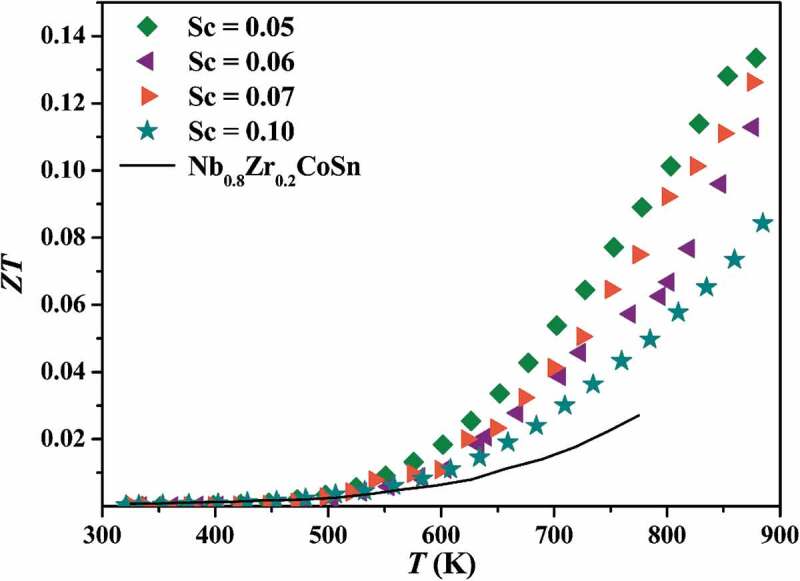
The figure of merit *ZT* for *p*-type NbCoSn and Nb_0.8_Zr_0.2_CoSn

Figure 10 shows the dimensionless figure of merit *ZT* of *p*-type samples. Due to the dramatic enhancement of power factor compared with *p*-type Nb_0.8_Zr_0.2_CoSn, and the significant suppression of thermal conductivity, the highest *ZT* of *p*-type Nb_0.95_Sc_0.05_CoSn achieves 0.13 at 879 K. It is much higher than that of Nb_0.8_Zr_0.2_CoSn [42], indicating Sc is an efficient *p*-type dopant for NbCoSn as compared to Zr.

## 4. Conclusions

In this work, homogenous Nb_1-*z*_Sc*_z_*CoSn compounds were prepared by an arc-melting process followed by an annealing treatment. The *p*-type NbCoSn compounds were obtained by substituting iso-electronic Sc at the Nb site and the effects on the electrical and thermal properties were investigated. Generally, the substitution of Sc at Nb site can change the *n*-type NbCoSn to a *p*-type semiconductor by adjusting the Fermi level, indicating Sc is an appropriate *p*-type dopant. Also, the thermal conductivity is reduced. As a result, the highest *ZT* is 0.13 at 879 K in *p*-type Nb_0.95_Sc_0.05_CoSn sample, which is roughly 5 times higher than that of *p*-type Nb_0.8_Zr_0.2_CoSn.

## Supplementary Material

Supplemental Material
