# Supplementary material for: Realizing p-type NbCoSn half-Heusler compounds with enhanced thermoelectric performance via Sc substitution
Source: Sci Technol Adv Mater. 2020 Feb 25;21(1):122–30. doi: 10.1080/14686996.2020.1726715 (PMC7054941; doi:10.1080/14686996.2020.1726715)
Supplement: Supplemental Material [file TSTA_A_1726715_SM8028.docx]

SUPPLEMENTARY INFORMATION FOR:

**Realizing *p*-type NbCoSn half-Heusler compounds with enhanced thermoelectric performance via Sc substitution**

Ruijuan Yan^a^, Wenjie Xie^a*^, Benjamin Balke^b^, Guoxing Chen^a^, Anke Weidenkaff^a,b^

^a^Technische Universität Darmstadt, Department of Materials Science, Alarich-Weiss-Straße 2, 64287 Darmstadt, Germany

^b^Fraunhofer Research Institution for Materials Recycling and Resource Strategies IWKS, Rodenbacher Chaussee 4, 63457, Hanau, Brentanostraße 2a, 63755 Alzenau, Germany

*Corresponding Author

E-mail: [wenjie.xie@mr.tu-darmstadt.de](mailto:wenjie.xie@mr.tu-darmstadt.de)

**Table S1** Physics parameters values: Debye temperature ***θ*_D_**, the sound velocity ***ν_s_***, Grüneisen parameter ***γ***, deformation potential ***E_def_***, density of state ***m*_d_^*^**, the longitudinal sound velocity ***ν_l_*** and grain size ***d*** for transport properties calculation.

| Parameters | Value | References |
| --- | --- | --- |
| *θ*_D_ | 361 K | [1] |
| *ν_s_* | 3141 m/s | [1] |
| *γ* | 1.2 | * |
| *E_def_* | 4.47 | [2] |
| *m*_d_^*^ | 3.4 | ** |
| *ν_l_* | 4956 m/s | * |
| *d* | 100-500 *μ*m | Figure S1 |

* fitting data ** calculated data

**Table S2** The average atomic mass of the crystal ***M*** and the average volume/atom ***Ω*** of Nb_1-_*_z_*Sc*_z_*CoSn.

| Compositions | *M* | *Ω* (10^-30^ m^3^/atom) |
| --- | --- | --- |
| Nb_0.99_Sc_0.01_CoSn | 90.02 | 70.50 |
| Nb_0.97_Sc_0.03_CoSn | 89.70 | 70.52 |
| Nb_0.96_Sc_0.04_CoSn | 89.54 | 70.50 |
| Nb_0.95_Sc_0.05_CoSn | 89.38 | 70.60 |
| Nb_0.94_Sc_0.06_CoSn | 89.22 | 70.70 |
| Nb_0.93_Sc_0.07_CoSn | 89.06 | 70.70 |
| Nb_0.90_Sc_0.10_CoSn | 88.58 | 71.20 |


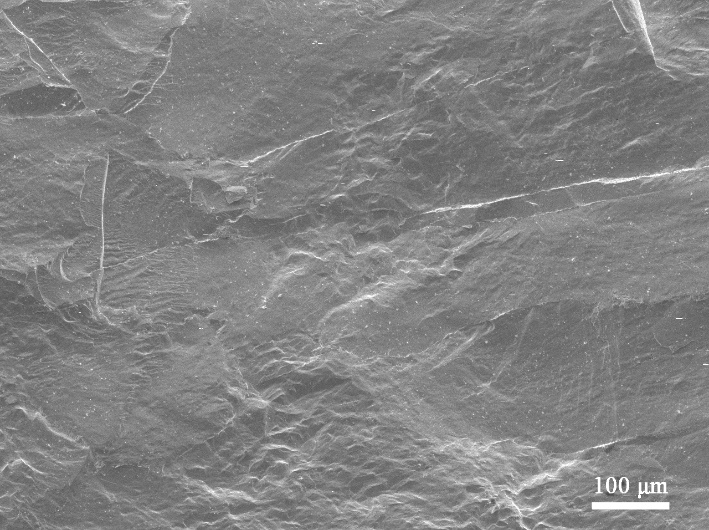


**Figure S1** Typical scanning electron microscopy image of Nb_0.93_Sc_0.07_CoSn.

**References**

[1] Ferluccio D A, Smith R I, Buckman J, et al. Impact of Nb vacancies and p-type doping of the NbCoSn-NbCoSb half-Heusler thermoelectrics. Phys Chem Chem Phys. 2018;20:3979-3987.

[2] Zhou J, Zhu H, Liu T-H, et al. Large thermoelectric power factor from crystal symmetry-protected non-bonding orbital in half-Heuslers. Nat Commun. 2018;9:1721.
